# Investigating the prospective associations of childhood sleep problems with persistent depression in emerging adulthood: the mediating role of inflammation

**DOI:** 10.1007/s00787-026-03053-z

**Published:** 2026-05-18

**Authors:** R. Amos, B. B. Durdurak, A. M. Gregory, S. Marwaha, I. Morales-Muñoz

**Affiliations:** 1 The Barberry, Severe Mood Disorders Clinic, Birmingham & Solihull Mental Health Foundation Trust, National Centre for Mental Health, Birmingham, UK; 2 Institute for Mental Health, School of Psychology, University of Birmingham, Birmingham, B15 2TT UK; 3 Department of Psychology, Royal Holloway, University of London, Egham Hill, Egham, UK

**Keywords:** Sleep duration, Chronotype, Inflammation, Depression, ALSPAC

## Abstract

Sleep problems constitute a risk for depression. However, it is unclear if persistent sleep problems in childhood are associated with enduring depression symptoms in emerging adulthood and the mechanisms underlying this. Data from the Avon Longitudinal Study of Parents and Children birth cohort were used to examine associations between persistent sleep problems across childhood and persistent depression across adolescence and emerging adulthood, and whether inflammation mediates this link. Predictors were parental report of the child’s nighttime sleep duration and midpoint of sleep (MPS), from 6 months to 6–7 years old. Our outcome was self-reported persistent higher depression symptoms, between 13 and 22 years. Latent Class Growth Analysis (LCGA) was used to detect trajectories for sleep duration, MPS, and depression symptoms and used in subsequent logistic regression analyses. Path analyses explored whether C-Reactive Protein [CRP] and Interleukin-6 [IL-6] at age 9 mediated the relationships between exposures and outcome. Data were available on 6,785 participants. Three separate LCGAs revealed distinct trajectories of interest: (a) persistent shorter nighttime sleep, (b) persistent later MPS, and (c) persistent higher depression symptoms. Children with persistent shorter nighttime sleep were at higher risk of persistent higher depression (OR = 1.94, 95%CI = 1.01–3.73, *p* = 0.046) and was partially mediated by increased IL-6 levels (bias-corrected estimate = 0·002, 95%CI = 0.001–0.003, *p* < 0.001). Persistent shorter nighttime sleep duration across childhood is longitudinally associated with persistent higher depression in adolescence into emerging adulthood, and inflammation is part of the mechanism. Addressing short sleep duration and inflammatory responses in childhood may prevent the development of depression in young people.

**Supplementary Information:**

The online version contains supplementary material available at https://doi.org/10.1007/s00787-026-03053-z.

## Introduction

Depression is a leading cause of disability, imposing substantial economic, societal, and individual burdens [1]. The adverse effect of depression on health can be profound, especially when symptom presentation is severe, recurrent, and/or chronic [2]. Indeed, those who exhibit repeated episodes or more chronic depression are less likely to respond to treatment, and the severity of the episodes tends to increase over time [2]. Depressive symptoms often emerge from 13 to 15 years, yet their developmental course is highly heterogeneous [3]. We have previously shown that a particularly at-risk subgroup exhibits persistent depression across adolescence [4], placing them at particularly high risk for poor long-term outcomes [5]. Identifying early-life factors that contribute to these more severe trajectories is therefore critical for informing timely and targeted early intervention strategies.

Sleep disturbances are a potential early marker of depression. Research has indicated that as many as 90% of depressed populations report sleep problems, with increasing evidence that sleep problems precede depressive episodes [6]. Two sleep problems related to depression that deserve special attention are short sleep duration and behavioural variations in circadian rhythms, such as chronotype (i.e., interindividual differences in daily activity patterns and sleep-wake cycles), as these are considered core sleep symptoms in depression [7]. Short sleep duration is prospectively associated with an increased risk of future depression in adult populations [6], and a late chronotype (i.e., preference for eveningness) is associated with subsequent depression symptoms, but not vice versa [8]. In adolescents, the relationship between chronotype and depression appears to be bidirectional [9], though longitudinal data remain limited [10]. Furthermore, longitudinal studies on short sleep duration and late chronotype have often relied on short follow-ups or adulthood outcomes at a single time point, leaving the long-term effects of early sleep patterns on depression insufficiently explored [11]. Of note, chronotype reflects a normal inter-individual variation in circadian timing and is not inherently pathological [12]; however, a late chronotype may increase vulnerability to depression [13, 14], particularly during developmental periods characterised by biological and social circadian misalignment [9, 15], potentially due to other factors such as lack of sleep due to incongruence between diurnal preference and societal demands to wake up early [12].

Sleep, however, is a multidimensional construct, and several sleep characteristics beyond duration and timing, including but not limited to variability in sleep timing and duration, reduced circadian rhythmicity, excessive intermittent awakenings, deficit in slow wave sleep, and insomnia symptoms, have also been linked to depression [16–18]. In the present study, we focused specifically on sleep duration and sleep timing (i.e., chronotype) for several reasons. First, sleep duration and circadian timing represent core dimensions of sleep–wake regulation and are closely linked to underlying circadian and homeostatic processes, which are hypothesised to play a central role in depression risk [19–21]. Second, both short sleep duration and late chronotype are highly prevalent in depression and have been associated with depressive symptoms across developmental stages [22–25]. Third, prior work from our group identified persistent shorter night-time sleep duration and late chronotype as distinct developmental sleep problems associated with poorer mental health outcomes [26–28], motivating further investigation of these patterns in relation to depressive symptom trajectories. Together, these considerations support our focus on sleep duration and timing as developmentally salient and clinically relevant indicators of depression risk.

Importantly, sleep and circadian rhythm disturbances are also strongly implicated in depression with onset prior to adulthood [29]. Disturbed sleep is highly prevalent among children and adolescents with depression [30] and is associated with higher depressive symptoms [29]. In a large clinical sample of children with major depressive disorder (MDD), those with sleep disturbance were more severely depressed than those without sleep problems, with the most severe symptom profiles observed among children presenting with both insomnia and hypersomnia [30]. Further, a meta-analysis in children and youth demonstrated that disturbed sleep is associated with depressive symptoms and increased odds of depression in prospective studies [31]. Together, these findings suggest that sleep and circadian disturbances are not merely correlates of adult depression but may be salient features of depressive risk earlier in development.

Beyond single time-point associations, longitudinal and trajectory-based studies indicate that trajectories of sleep difficulties across childhood may be particularly informative for later internalising problems. For example, low sleep rhythmicity (i.e., irregularities in sleeping schedules in early childhood), has been shown to predict adolescent-onset MDD and anxiety disorders [32]. More recent work using trajectory modelling has further demonstrated that children following a long sleep latency trajectory across childhood exhibit higher levels of depressive symptoms in adolescence [33]. Similar evidence from prospective cohort studies suggests that early sleep problems, including prolonged sleep onset latency and refusal to sleep alone are associated with greater MDD and anxiety severity later in development [34]. Collectively, these findings underscore the importance of considering the persistence and developmental patterns of sleep disturbances, rather than isolated exposures, when examining pathways to later depression.

Despite established links between sleep problems and later depression in young people, the physiological mechanisms mediating this relationship remain unclear. Among potential physiological mediating factors, inflammation may be one biological pathway linking sleep disruption to depression [35–38]. Evidence indicates that persistent sleep disturbance and shorter sleep duration are associated with higher levels of peripheral inflammatory markers, such as interleukin-6 (IL-6) and C-reactive protein (CRP) [39, 40], which have also been implicated in depression risk [41–44]. Peripheral inflammatory activity may influence mood through immune-to-brain communication via humoral and neural pathways, with downstream effects on glial activation and neurobiological processes implicated in depression [35]. For example, a longitudinal study of adults aged 50 years and older found that greater sleep disturbance at baseline was associated with higher mid-assessment serum CRP, which in turn significantly mediated the association between self-reported sleep disturbance and depressive symptom severity at follow-up in women [45]. Supporting these findings, animal models also show that sleep disturbance can induce depressive-like behaviours alongside neuroinflammatory changes, including microglial and astrocytic activation in the anterior cingulate cortex and increased pro-inflammatory cytokines [46, 47]. Together, these findings suggest that sleep disturbance may contribute to depression by increasing the levels of inflammation. However, research testing this mediation pathway remains very limited [35], particularly in young people given that early life and childhood may represent sensitive developmental windows during which sleep disruption can have disproportionate effects on immune regulation and brain development, potentially increasing vulnerability to later depressive symptoms [48].

To address existing gaps, the primary aim of our study was to investigate whether persistent poor sleep (i.e., short sleep, late chronotype) across childhood is prospectively associated with persistent depressive symptoms from adolescence into emerging adulthood, and whether this relationship is mediated by inflammatory markers. We hypothesised that a persistent pattern of short sleep duration and late chronotype across childhood would increase the risk of persistent depression symptoms, and that elevated inflammation would mediate these relationships.

## Methods

### Participants

The Avon Longitudinal Study of Parents and Children (ALSPAC) is a UK birth cohort study examining the determinants of development, health, and disease during childhood and beyond [49, 50]. Pregnant women who were residents of Avon, UK, with expected dates of delivery between April 1, 1991, and December 31, 1992, were invited to take part. The initial number of pregnancies enrolled was 14,541. Of these, there were a total of 14,676 foetuses. When the cohort was approximately 7 years, the sample was bolstered. The total sample size for analyses using child-based data collected after the age of 7 therefore includes 15,454 pregnancies resulting in 15,589 known foetuses. Twins and multiple pregnancies were not included in analyses. We followed the Strengthening the Reporting of Observational Studies in Epidemiology (STROBE) reporting guidelines [51].

## Measures

### Nighttime sleep duration across childhood

The duration of the child’s nighttime sleep was parent-reported. Parents were asked what time their child normally went to sleep at night and awoke in the morning. The difference between these two timepoints was used to derive nighttime sleep duration. This information was collected at 6, 18, and 30 months, and at 3.5, 4–5, 5–6, and 6–7 years.

## Midpoint of sleep across childhood

Midpoint of sleep (MPS) is a proxy of chronotype, which is the middle time point between bedtime and wake-up time. MPS is highly correlated with dim-light melatonin onset [52] and is a reliable marker of chronotype in children [53]. Bedtime and wake-up time were parent-reported at 6, 18, and 30 months, and at 3.5, 4–5, 5–6, and 6–7 years.

## Inflammation at 9 years

CRP and IL-6 were selected as inflammatory markers because they are well-established indicators of low-grade systemic inflammation and have been widely used in epidemiological studies of mental health [54–56]. Both markers have been measured reliably and repeatedly within ALSPAC, with validated assay protocols [57]. Importantly, CRP and IL-6 have been extensively examined in relation to depressive symptoms [58–61] and related psychopathology [62–64] within ALSPAC and comparable population-based samples, including in prior work by our group [27, 28, 65]. Selecting these markers therefore allowed us to build on existing longitudinal research within the cohort and to maximise comparability with previous findings linking inflammation to depression risk.

Serum IL-6 and CRP levels were assessed in non-fasting blood samples collected at 9 years of age, during a clinic visit [60, 66]. Samples were immediately spun and stored at − 80 °C for a median 7.5 years with no evidence of freeze–thaw cycles during storage. CRP was analysed using an automated particle-enhanced immunoturbidimetric assay (Roche UK, Welwyn Garden City, UK), while interleukin-6 (IL-6) was assessed via a single enzyme-linked immunosorbent assay (R&D Systems, Abingdon, UK). The minimum detection limit was 0.007 pg/ml and 0.01 mg/l for IL-6 and CRP, respectively. IL-6 and CRP levels were log-transformed to adjust for a positive skew. All inter-assay coefficients of variation were less than 5%.

### Depression symptoms across adolescence and emerging adulthood

The Short Mood and Feelings Questionnaire (SMFQ) [67] was used to assess self-reported depression symptoms at 12.5, 13.5, 16, 17.5, 21, and 22 years (see Supplementary Table S1). SMFQ is a well-validated 13-item questionnaire inquiring about the occurrence of depression symptoms over the past 2 weeks [68–71]. We used the SMFQ total score, with higher scores indicating greater depression symptoms (12 > indicates depression). SMFQ is a widely used measure in young people and was chosen from other measures in ALSPAC as it included self-report data across adolescence and into adulthood [71]. It evidences high psychometric validity in young people and discriminates between those with MDD cases from non-cases to a high degree of accuracy [71].

## Covariates

Covariates were selected a priori based on established evidence linking early-life socio-economic and familial adversities to both sleep development [72] and later depression [73, 74], and in line with prior cohort studies examining sleep and mental health outcomes using ALSPAC data [28, 75, 76]. We focused on covariates measured during the perinatal period and early years of life, given evidence that exposures during these sensitive developmental windows can have enduring effects on sleep regulation and mental health across the life course [77, 78]. Specifically, covariates included child’s sex at birth given the higher prevalence of depression in females [79] child’s birth weight (in kg), due to associations between low birth weight and later depression risk [80] ethnicity, as depression prevalence varies across ethnic groups [81], as well as family adversity (e.g. housing, family conditions, maternal psychopathology ), given its association with offspring depression [82], and maternal socioeconomic status, given that lower social class has been associated with depression [73, 83]. In path analyses, Body Mass Index (BMI) at 9 years was also entered as a covariate, given that higher BMI is related to higher levels of inflammation [84]. A detailed description of covariates and their measurement timing is provided in Supplementary Table S1.

### Statistical analysis

#### Latent class growth analyses

We modelled sleep trajectories by conducting three separate Latent Class Growth Analyses (LCGA) of firstly sleep duration, secondly MPS, and finally depression symptoms using Mplus8. This was to enable derivation of latent classes and trajectories for each variable of interest.

We conducted two separate LCGAs to model trajectories of parent-reported nighttime sleep duration and MPS at 6, 18, and 30 months, and at 3.5, 4–5, 5–6, and 6–7 years. Then, we conducted a third separate LCGA where we modelled trajectories of depression symptoms at 12.5, 13.5, 16, 17.5, 21, and 22 years of age. Starting with a single k-class model, k + 1 solutions were fitted until the optimum model was reached [85, 86]. The optimal classification model was determined using the following parameters: Bayesian Information Criteria (BIC), significant Vuong-Lo-Mendell-Rubin (VLMR), entropy value, the proportion of individuals in each trajectory (> 3%), and the interpretability of each class [85]. LCGAs were conducted using all available repeated measured data. Participants were included in the LCGA if they contributed at least one valid measurement at any assessment wave. Given the longitudinal nature of ALSPAC, varying levels of attrition occurred across measurement waves. We therefore quantified the proportion of missing values at each assessment included in the trajectory models. For nighttime sleep duration, missingness ranged from 28.3% at 6 months to 48.4% at 6–7 years. For MPS, missingness ranged from 29.9% to 48.4% across the same period. For depression symptoms, missingness ranged from 57.1% at age 12.5 years to 78.9% at age 21 years (see Supplementary Tables S5–S7 for full details). Missing values due to attrition were handled by the full information maximum likelihood estimation (FIML) method [85]. FIML incorporates all available observations under the Missing At Random assumption and allows participants with partial data across waves to contribute to trajectory estimation, thereby minimising bias due to intermittent missingness [87–90].

We followed the Guidelines for Reporting on Latent Trajectory Studies (GRoLTS) throughout (see Table S8 in Supplement) [91].

#### Regression analyses

Regression and path analyses were restricted to participants with complete class assignment for both sleep and depressive symptom trajectories. These analyses additionally required availability of the relevant inflammatory markers and covariates. As a result, the sample size varied across models depending on data availability for each outcome and mediator. Using the classes identified by LCGA, we conducted two separate binary logistic regressions to explore the prospective association of nighttime sleep duration and MPS with persistent depression symptoms at 13–22 years of age. The derived latent classes from nighttime sleep duration and MPS LCGA models were entered as predictors in each model, respectively, and the normative class (as indicated by the largest sample size) was used as the reference variable. The outcome variable was derived from the depression LCGA classes. In line with previous methods [28], this variable was dichotomised, where 1 = persistent higher depression symptoms class and 0 = the normative class (as indicated by larger sample size). We conducted unadjusted and adjusted models and controlled for all covariates listed above, apart from BMI, which was only included as a covariate for the mediation analyses.

Additionally, sensitivity analyses were conducted using multinomial regressions to explore whether nighttime sleep duration and MPS showed differing associations by each of the differential depression trajectories, rather than only with two of them. Two separate multinomial models were run, firstly with nighttime sleep duration and secondly with MPS as the predictors.

Although FIML addresses missingness within the LCGA models, we additionally utilised inverse probability weighting (IPW) method to account for selection bias in the subsequent logistic regression analyses. To do this, we first identified variables associated with dropout by fitting logistic regression models predicting non-response at the first SMFQ assessment (age 12.5 years; see Supplementary Table S2). All variables significantly associated with attrition were then included as predictors in a logistic model with response status (1 = responded at age 12.5 years; 0 = non-response) as the outcome. From this model, we computed IPWs for each participant, defined as the inverse of the predicted probability of response. These weights were applied in all regression analyses to reduce bias arising from selective dropout. This approach aligns with previous research using ALSPAC [5, 92, 93].

Finally, we examined whether increased levels of inflammation (IL-6 and CRP) at 9 years mediated the relationship between persistent poor sleep trajectories (from 6 months to 7 years) and persistent depression symptoms (from 13 to 22 years). We conducted two separate path analyses, one for each inflammatory marker, in SPSS AMOS 26. We controlled for sex, age of mother, and FAI, as these were the most relevant covariates as indicated from the logistic regression analyses. We also controlled for BMI at 9 years. All models were bootstrap bias corrected.

## Results

Of the total sample, 49% were female, and 5.1% were of an ethnicity other than white (see Table 1 for all descriptive results). For sleep trajectories, the available sample was 12,392. For depression trajectories, data were available for 6,785 participants, and this was the total sample used in our primary analyses.

**Table 1:** Descriptive statistics, sociodemographic variables, sleep duration, MPS, inflammatory markers and depression of the whole ALSPAC sample*

Variable	Mean (SD)	*N* (%)
Sociodemographic/other covariates		
Sex		
Female	-	7337 (48.9)
Male	-	7664 (51.1)
Ethnicity		
White	-	11,520 (94.9)
Other	-	613 (5.1)
Birth weight, kg	3.38 (0.58)	-
Age of mother at delivery	27.99 (5.00)	-
FAI, total score	4.28 (4.44)	-
CAMSIS	52.76 (13.59)	-
Nighttime sleep duration (hours)		
6 months	10:99 (2:30)	-
18 months	11:33 (1:08)	-
30 months	11:24 (1:01)	-
3.5 years	11:28 (0:89)	-
4–5 years	11:39 (0:72)	-
5–6 years	11:28 (0:72)	-
6–7 years	11:00 (0:64)	-
MPS (hours)		
6 months	1:55 (0:81)	-
18 months	1:51 (0:79)	-
30 months	1:45 (0:69)	-
3.5 years	1:40 (0:62)	-
4–5 years	1:44 (0:53)	-
5–6 years	1:52 (0:51)	-
6–7 years	1:90 (0:53)	-
Depression symptoms (SMFQ total)		
12·5 years	3.97 (3.86)	-
13·5 years	4.92 (4.49)	-
16 years	5.91 (5.64)	-
17·5 years	6.58 (5.25)	-
18 years	6.82 (5.93)	-
21 years	5.70 (5.58)	-
22 years	6.20 (5.53)	-
Inflammatory markers		
CRP at 9 y, mg/L	0.83 (2.72)	-
IL-6 at 9 y, mg/L	1.29 (1.59)	-

*SMFQ* Short Mood & Feelings Questionnaire; a score of 12 or above is indicative of depression [31]. *FAI* Family adversity index score ranges from 0–18, with higher scores indicating more family adversity [49]. *CAMSIS* Cambridge social interaction and stratification scale (range = 0-100), scores are standardised on a scale from 0 to 100 with a mean of 50 and a standard deviation of 15. A higher score indicates higher positions in the social hierarchy, such as higher income, educational attainment and stronger connections to those in higher status positions [40]. *CRP* C-Reactive Protein, IL-6 = Interleukin-6. *MPS* Midpoint of sleep, A lower score shows a preference for earlier sleep and earlier waking (lark-like), whilst a higher score shows a preference for later sleeping (owl-like)

* Table 1 describes the full ALSPAC core sample. Analytic sample sizes for each analysis are reported in the Results section and differ due to outcome-specific data availability and attrition

### Nighttime sleep duration trajectories

The night-time sleep duration LCGA included 12,393 participants with at least one sleep duration assessment between 6 months and 6–7 years; 3,252 participants were excluded from this LCGA because they had missing data on all night-time sleep duration indicators.

**Fig. 1:**
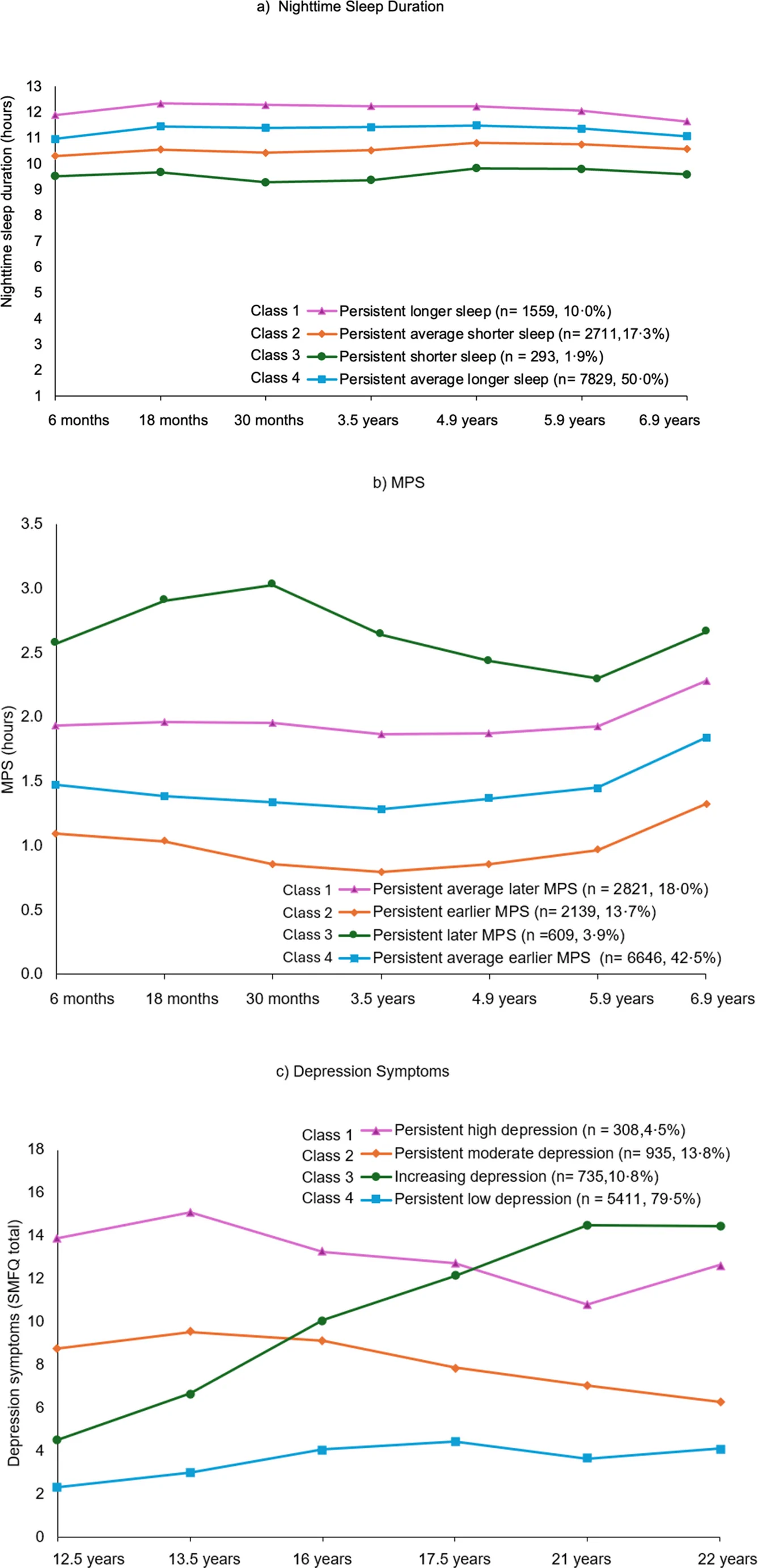
Trajectories identified by Latent Class Growth Analysis for **a**) nighttime sleep duration **b**) Midpoint of sleep (MPS) and **c**) depression symptoms. *Note*: A four-class solution evidenced best fit across all three Latent Class Growth Analyses. Panel (**a**) illustrates the four longitudinal trajectories of sleep duration throughout childhood, where the mean number of parent-reported hours slept is presented at each time point for each class. Class 1 represents children who exhibited a pattern of persistent longer sleep across timepoints. Class 2 represents children who exhibited a pattern of persistent average shorter nighttime sleep. Class 3 represents children with a pattern of persistent short sleep over time points and was the first predictor of interest in this study. Class 4 represents children with a pattern of persistent average longer sleep. Panel (**b**) represents the four longitudinal trajectories of MPS, again, the mean number of hours of MPS is presented at each time point for each class. Class 1 represents children with a persistent average later MPS across time points. Class 2 represents children with a persistent earlier MPS (more lark-like). Class 3 represents children with a persistent later MPS and was the second main predictor of interest in this study. Class 4 represents children with a pattern of persistent average earlier MPS over time points. Panel (**c**) illustrates the four longitudinal trajectories of depression symptoms over adolescence and into emerging adulthood. The mean total SMFQ score is presented at each time point. Class 1 represents individuals with a pattern of persistent higher depression symptoms and was the main outcome of interest in this study. Class 2 represents individuals with a pattern of persistent moderate depression symptoms. Class 3 represents individuals with a pattern of increasing depression over time points and class 4 represents those with persistent low depression symptoms

For nighttime sleep duration, a 4-factor solution was chosen, given excellent fit (i.e., a VLMR *p* value of < 0.001) and having the highest entropy and the lowest BIC value out of the significant models (see Table 2). Class 1 was characterized by persistent longer sleep duration (*n* = 1559, 10.0%); class 2 by persistent average shorter nighttime sleep (*n* = 2711, 17.3%); class 3 by persistent shorter nighttime sleep (*n* = 293, 1.9%) and class 4 by persistent average longer nighttime sleep (*n* = 7829, 50.0%) (See Fig. 1a).

**Table 2:** Bayesian information criteria (BIC), Vuong–Lo–Mendell–Rubin (VLMR) likelihood test *p*-values and entropy for Classes 2–6 for nighttime sleep duration, MPS and depression scores

Nighttime sleep duration from 6 months to 7 years			
	BIC	VLMR *P*-value	Entropy
1 class	193265.720	---	---
2 classes	173960.109	< 0.001	0.750
3 classes	171052.404	< 0.001	0.769
4 classes	**170000**.**824**	**< 0**.**001**	**0**.**788**
5 classes	169365.553	0.232	0.683
6 classes	169324.312	0.332	0.711
MPS from 6 months to 7 years			
	BIC	VLMR *P*-value	Entropy
1 class	131338.155	---	---
2 classes	119423.667	< 0.001	0.690
3 classes	114795.864	< 0.001	0.714
4 classes	**113182**.**568**	**< 0**.**001**	**0**.**741**
5 classes	112113.789	0.146	0.686
6 classes	112489.669	0.935	0.530
Depression scores (SMFQ) from 12 years to 22 years			
	BIC	VLMR *P*-value	Entropy
1 class	183761.391	---	---
2 classes	171931.914	< 0.001	0.810
3 classes	170300.869	< 0.001	0.776
4 classes	**169309.033**	**< 0.001**	**0**.**762**
5 classes	168842.632	< 0.001	0.759
6 classes	168580.590	0.056	0.715

*MPS* Midpoint of sleep, *SMFQ* short mood and feelings questionnaire

The chosen solution is in bold

No external cut-off values were applied to define “shorter” or “longer” sleep duration. Instead, these labels reflect relative differences between the latent trajectory groups identified by the LCGA, such that trajectories with consistently shorter nighttime sleep duration across childhood were classified as “shorter” relative to trajectories with longer sleep duration within the sample. Thus, trajectory group membership was entirely data-driven and determined by the LCGA model rather than by comparison to predefined thresholds or normative values.

### MPS trajectories

The MPS LCGA included 12,216 participants with at least one MPS assessment between 6 months and 6–7 years; 3,429 participants were excluded from this LCGA because they had missing data on all MPS indicators.

Out of multiple class factor solutions, a 4-factor solution was chosen given its excellent fit with a VLMR *p* value of < 0.001 and having the highest entropy and the lowest BIC value out of the significant models (see Table 2). Class 1 was characterised by persistent average later MPS (*n* = 2821, 18.0%), class 2 by persistent earlier MPS (*n* = 2139, 13.7%), class 3 by persistent later MPS (*n* = 609, 3.9%) and class 4 by persistent average earlier MPS (*n* = 6646, 42.5%) (See Fig. 1b).

No external cut-off values were applied to define “earlier” or “later” chronotype. Instead, these labels reflect relative differences between the latent MPS trajectory groups identified by the LCGA, such that trajectories with consistently earlier MPS values across childhood were classified as “earlier” relative to trajectories with later MPS values within the sample. Thus, chronotype groups were entirely data-driven and determined by the LCGA model rather than by comparison to predefined or normative thresholds.

### Depression symptoms trajectories

The SMFQ LCGA included 8,771 participants with at least one SMFQ assessment between ages 12 and 23 years; 6,874 participants were excluded from this LCGA because they had missing data on all SMFQ indicators.

Although the five-class model showed the lowest BIC, the 4-class model had a higher entropy value. This suggests that a four-class model provided better classification accuracy than the 5-class model. Additionally, the 4-class model maintained adequately large group sizes (e.g., exceeding 3%) for each class [85]. The 4 classes were persistent higher depression symptoms (class 1, *n* = 308, 4.5%), persistent moderate depression symptoms (class 2, *n* = 735, 10.8%), increasing depression (class 3, *n* = 935, 13.8%) and persistent lower depression symptoms (class 4, *n* = 5411, 79.5%) (See Fig. 1c).

### Prospective associations between sleep duration and MPS trajectories with persistent depression symptoms

The association between night-time sleep duration trajectories and persistent depressive symptoms was examined in 1,756 participants with complete data on sleep trajectory class, persistent higher depression symptoms class, and covariates. Logistic regressions revealed that shorter nighttime sleep duration from 6 months to 7 years was prospectively associated with persistent higher depression between 13 and 22 years in adjusted models (OR = 1.94, 95%CI = 1.01, 3.73, *p* = 0.046).

The association between MPS trajectories and persistent depressive symptoms was examined in 1,767 participants with complete data on MPS trajectory class, persistent higher depression symptoms class, and covariates. There was no significant association between MPS trajectories and persistent higher depression in adjusted models. See Table 3 for all results.

**Table 3:** Logistic regression associations between latent classes of sleep duration and MPS, with persistent higher depression symptoms

	Unadjusted model		Adjusted model	
	OR (95% CI)	*P* value	OR (95% CI)	*P* value
*Nighttime sleep duration*				
Class 3 (persistent shorter nighttime sleep)	2.08 (1.11, 3.89)	0.022	1.94 (1.01, 3.73)	0.046
Class 2 (persistent average shorter nighttime sleep)	1.01 (0.76, 1.34)	0.959	1.10 (0.82, 1.48)	0.511
Class 1 (persistent long nighttime sleep)	1.34 (0.98, 1.85)	0.070	1.13 (0.81, 1.56)	0.483
Sex assigned at birth^a^			2.82 (2.19, 3.63)	< 0.001
Birth weight, kg			1.01 (0.82, 1.25)	0.927
Age of mother at delivery			0.97 (0.95, 1.00)	0.046
Ethnicity^b^			0.67 (0.35, 1.27)	0.219
FAI, total score			1.09 (1.06, 1.11)	< 0.001
CAMSIS score			1.00 (0.99, 1.01)	0.359
*MPS*				
Class 3 (persistent later MPS)	1.49 (0.90, 2.49)	0.124	1.16 (0.68, 1.97)	0.582
Class 2 (persistent earlier MPS)	0.93 (0.68, 1.28)	0.674	1.02 (0.74, 1.41)	0.884
Class 1 (persistent average later MPS)	1.33 (1.01, 1.75)	0.042	1.20 (0.91, 1.59)	0.206
Sex assigned at birth^a^			2.79 (2.17, 3.60)	< 0.001
Birth weight, kg			1.01 (0.82, 1.25)	0.923
Age of mother at delivery			0.98 (0.95, 1.00)	0.054
Ethnicity			0.65 (0.34, 1.24)	0.187
FAI, total score			1.09 (1.06, 1.11)	< 0.001
CAMSIS score			1.00 (0.99, 1.01)	0.403

Class 4 was the reference class for sleep duration and MPS. ^a^The reference value was male, ^b^the reference value was a white ethnicity. FAI = Family Adversity Index, a higher score indicates more adversity. CAMSIS score = Cambridge Social Interaction and Stratification larger scores indicate those with higher social class or occupational roles. MPS = Midpoint of sleep (a proxy of chronotype)

### Sensitivity analyses

**Fig. 2:**
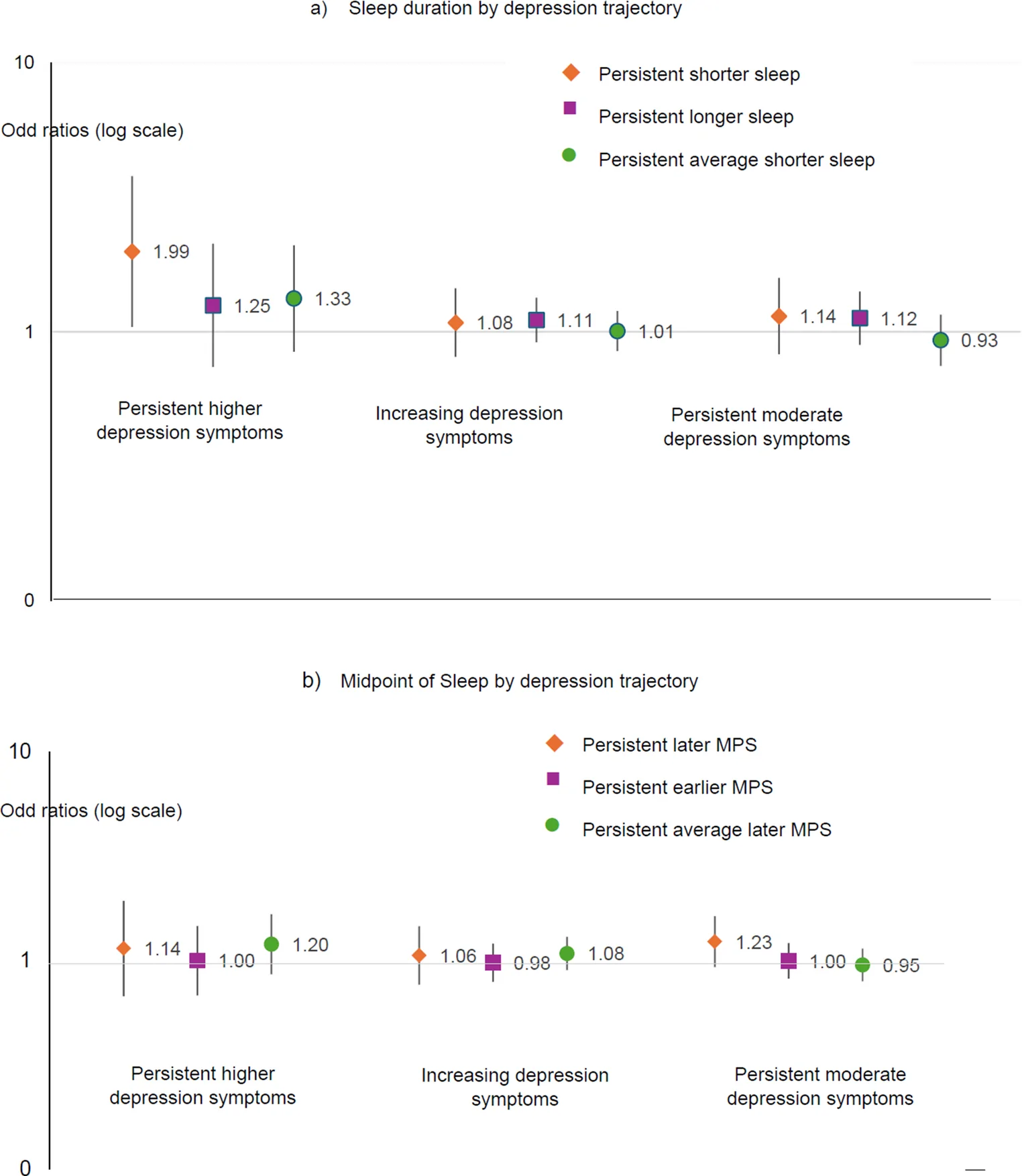
Odd ratios and 95% CIs from multinomial regressions for **a**) short sleep duration and **b**) Midpoint of sleep, by persistent higher depression, increasing depression and moderate depression (adjusted model). *Note*: * *p* < 0·05. Data points represent odd ratios, and horizontal intersecting lines represent 95% confidence intervals. This figure shows the relationship between (**a**) persistent shorter nighttime sleep, persistent longer sleep and persistent average short sleep and (**b**) Persistent later MPS, persistent earlier MPS and persistent average later MPS on persistent higher depression, increasing depression, and persistent moderate depression. The multinomial regression model was adjusted for sex, birthweight (kg), age of mother at delivery, ethnicity, family adversity score, and social class

Sensitivity analyses showed that persistent shorter nighttime sleep duration was prospectively associated with persistent higher depression (vs. persistent low depression, as the reference) in adjusted models (OR = 1.99, 95%CI = 1.04, 3.78, *p* = 0.037) (See Fig. 2a). However, we did not find any additional significant associations between the remaining nighttime sleep duration trajectories (i.e., longer sleep duration, average shorter sleep) and persistent higher depression, increasing depression and persistent moderate depression (see Fig. 2a, Supplementary Table S3). With MPS, there was no significant effect of any trajectories on any of the depression trajectories in adjusted models (see Fig. 2b, Supplementary Table S4).

### Path analyses

Path analyses including inflammatory markers as mediators were conducted in *N* = 4,038 (CRP model) and *N* = 4,046 (IL-6 model) participants with complete data.

**Fig. 3:**
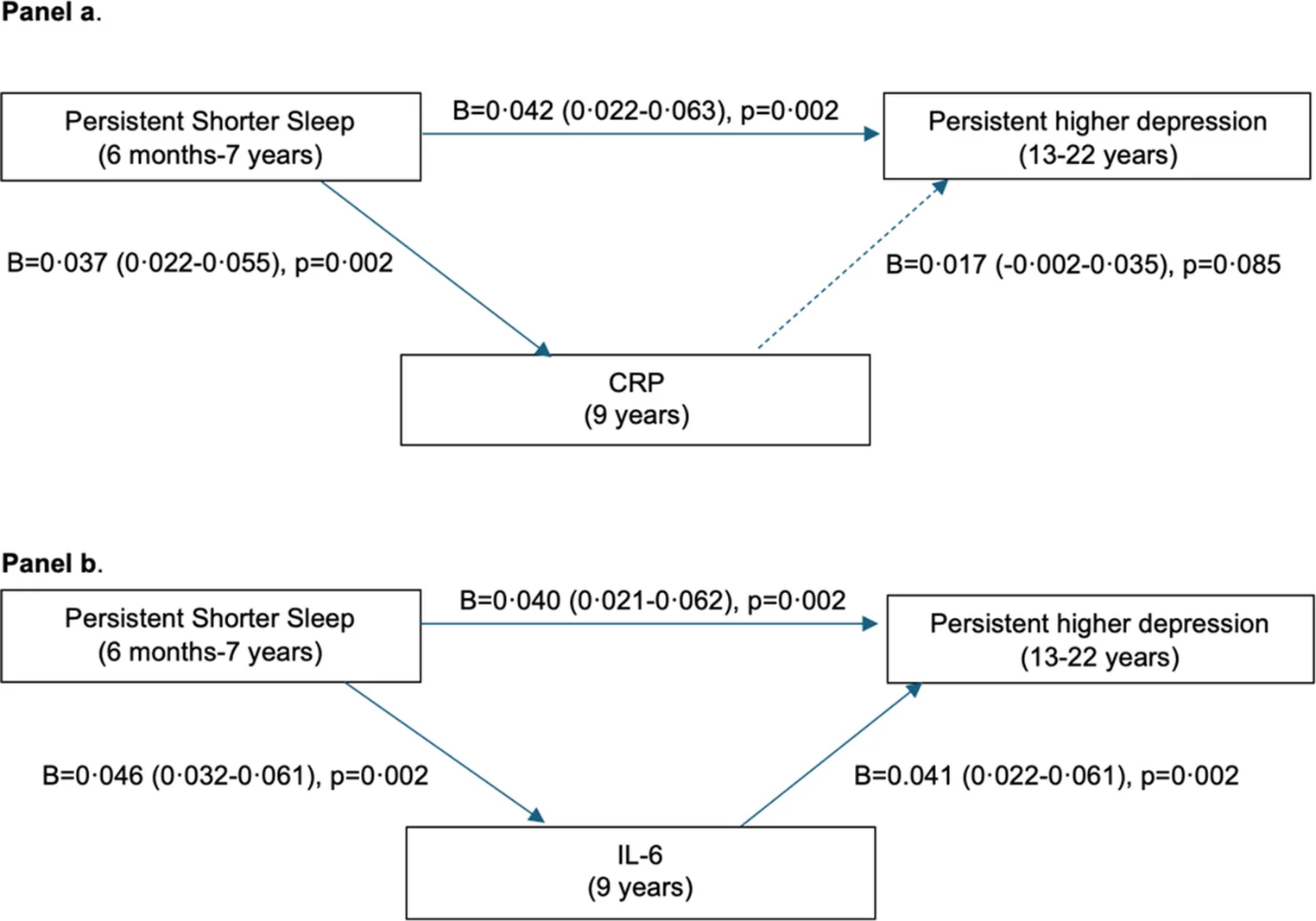
Path analyses of persistent shorter nighttime sleep, CRP and IL-6 on persistent higher depression. *Note*: The figure above depicts the direct associations of the independent, mediating, and dependent variables. Persistent shorter nighttime sleep duration represents the independent variable; C-reactive protein (CRP) (panel **a**), and Interleukin-6 (IL-6) (panel **b**) at 9 years represent the mediating factors, and persistent higher depression symptoms at 13–22 years represent the outcome. The covariates included in these path analyses were the Family Adversity Index, sex, and age of mother at delivery. Body mass index (BMI) at 9 years was also added as a control variable. Significant pathways are signified by solid arrows and nonsignificant pathways by dashed arrows

When examining whether CRP at 9 years mediated the prospective associations between persistent shorter nighttime sleep duration and persistent higher depression (i.e., as this was the main significant finding from the regression analyses), path analysis model fit indexes exhibited excellent model fit (χ^2^ = 2.369, *p* = 0.796, root mean square error of approximation [RMSEA] = 0.000, comparative fit index [CIF] = 1.000). However, there was no indirect significant effect of persistent short sleep on persistent higher depression symptoms via CRP (bias-corrected estimate = 0.001, 95%CI = 0.000-0.002, *p* = 0.057). The IL-6 model also exhibited an excellent model fit (χ^2^ = 2.331, *p* = 0.802, RMSEA = 0.000, CIF = 1.000). There was a significant indirect effect of persistent short sleep on persistent higher depression symptoms via IL-6 (bias-corrected estimate = 0.002, 95%CI = 0.001–0.003, *p* < 0.001). This indicates that those who exhibit persistent shorter nighttime sleep between 6 months − 7 years are more likely to have higher levels of IL-6 at 9 years, and this is associated with persistent higher depression between 13 and 22 years. See Fig. 3 for direct associations for each path analysis.

## Discussion

To our knowledge, this is the first study to demonstrate the detrimental effect of persistent shorter nighttime sleep duration from infancy to childhood on more enduring and severe forms of depressive symptoms across adolescence and emerging adulthood. It is also the first to explore the prospective association between sleep problems, inflammation and subsequent depression symptoms. We found mixed evidence regarding the mediating role of inflammation; whilst IL-6 partially mediated the relationship between persistent shorter nighttime sleep and persistent higher depression symptoms, CRP did not. Additionally, we did not find a significant relationship between MPS and persistent depression symptoms.

Our results show that children who experience shorter nighttime sleep duration from 6 months to 7 years are nearly twice as likely to exhibit a pattern of persistently high depression symptoms that persist from 13 to 22 years of age. Sensitivity analyses confirmed that this was specific to persistent depressive symptoms. These results are congruent with previous findings from longitudinal population cohorts where short sleep in adolescence increases the risk for MDD at 16 years old [94] and sleep problems (e.g. sleep less than other children) as early as 4 years, have a prospective relationship to depression symptoms in mid-adolescence [95]. Our study extends this evidence by highlighting that the persistence of short sleep duration may be a risk factor for persistent and higher levels of depression symptoms in the long term.

Contrary to our hypothesis, we found no association between later MPS and persistent higher depression. In adults with MDD, a later chronotype shows causal links to subsequent depression symptoms, but not vice versa [8]. In adolescents, the relationship between chronotype and depression appears to be bidirectional, and specifically, post-pubertal children with a prior history of depression show the greatest preference for eveningness [9]. However, important developmental differences may help explain the lack of association observed in the present study. Much of the existing chronotype literature has focused on adolescents and adults, developmental periods during which individuals exert greater autonomy over their sleep–wake schedules. In contrast, during infancy and early childhood, sleep timing is largely externally regulated, and apparent late sleep timing may reflect parental preferences, household routines, or broader contextual factors rather than a child’s intrinsic circadian preference [96–98]. As such, chronotype measures in early childhood may be less sensitive to biologically driven circadian variation. Accordingly, in the context of early childhood, later sleep timing may be interpreted as reflecting family and context-driven sleep patterns rather than an intrinsic child chronotype. Additionally, prior studies have typically examined chronotype over shorter follow-up periods in adolescence or adulthood [9], whereas our study assessed sleep timing trajectories from infancy through childhood. Moreover, the way we derived MPS is limited as it is an indirect measure of chronotype and is parent-reported. The possible relationship between chronotype and persistent higher depression should be explored using more objective measurements of chronotype such as actigraphy and polysomnography. Together, these developmental and methodological differences may account for the weaker or null associations observed for chronotype in the present analyses.

Finally, we found that the relationship between shorter nighttime sleep and persistent depressive symptoms was mediated by increased levels of IL-6 at 9 years of age, but not CRP. Although both are pro-inflammatory markers, IL-6 is an upstream, rapidly responsive cytokine that increases in reaction to acute stressors such as sleep disruption [99], whereas CRP is a downstream acute-phase protein that more strongly reflects cumulative inflammation and is heavily influenced by lifestyle and environmental factors [100, 101]. For example, previous cohort studies have shown that elevated IL-6 in late childhood is associated with later depression [57, 102], while CRP tends to be associated when inflammatory load increases proximally to the onset of depressive symptoms, as demonstrated by rising CRP trajectories in adolescence [103].

Cross-sectional research has also found elevated IL-6 to be linked to short sleep (< 6 h) in depressed patients [104]. Prior work by our group exploring prospective associations with sleep as a predictor has found IL-6 at 9 years to be related to ADHD at 10 years [27] and psychosis at 24 years [28], while CRP was found to mediate the association between persistent anxiety and psychosis at 24 [105]. As such, it appears that increased CRP is specific to anxiety [105] and increased IL-6 is specific to sleep [27, 93], at least in ALSPAC. These findings suggest that increased IL-6 is one possible physiological pathway by which persistent shorter nighttime sleep relates to enduring and more severe depression symptom presentation. However, further research is required to confirm whether IL-6 mediates this link across different samples.

Given our findings that short sleep duration across childhood is associated with persistent higher depression symptoms across adolescence and emerging adulthood, it is imperative to investigate early interventions for depression targeting sleep in paediatric populations. For children and adolescents presenting with depression symptoms, the National Institute for Health and Care Excellence (NICE) recommends psychological and family-based interventions [106]. The implementation of sleep interventions poses an economically viable, possibly less stigmatising and non-pharmacological approach to alleviating low-grade inflammation and mitigating future depression symptoms and is likely to be beneficial across psychiatric conditions.

This study’s strengths include a large population-based sample and longitudinal methodology, enabling exploration of persistent poor sleep, persistent higher depression, and the mediating role of inflammation. However, there are some limitations to be considered. First, sleep measures were parent-reported and objective measures such as actigraphy were unavailable. Although studies show that parental sleep reports are convergent with objective sleep measures [107], a combination of subjective and objective sleep measures is warranted to verify our results. In early childhood, sleep patterns are heavily influenced by parental lifestyles and behaviours which are important factors to account for in future research. Additionally, the derived sleep duration trajectories are based on nighttime sleep duration, and the influence of daytime napping behaviours were not explored. As such, we cannot account for total sleep duration, especially during the first years of life (e.g., from 6 months to 3.5 years), as daytime sleep duration was unavailable. Furthermore, as mentioned before, sleep is a multidimensional construct, and other sleep characteristics, such as insomnia symptoms, sleep variability, and circadian rhythmicity, have also been implicated in depression risk [16–18]. In the present study, our focus on sleep duration and sleep timing was driven in part by the availability of harmonised measures across multiple waves of childhood, which enabled longitudinal trajectory modelling. Future studies should extend this work by examining developmental trajectories of other sleep dimensions, to provide a more comprehensive understanding of how sleep development shapes risk for depression.

Second, we relied on self-reported depression symptoms assessed via the SMFQ. Although SMFQ has been validated in adolescents [71], self-reported SMFQ items are not fully reflective of diagnostic criteria.

Third, although emerging evidence suggests that sleep difficulties precede depression [6] the associations we have found between short sleep duration and persistent higher depression are not necessarily causal, and the direction of effects may be bidirectional. Moreover, it is worth noting we did not account for depression prior to 12.5 years of age and cannot rule out prior depression symptoms before this age and their impact on our findings. This limitation is particularly noteworthy given evidence that depression and subthreshold depressive symptoms can first emerge in childhood and early adolescence [1, 108], with a substantial proportion of lifetime cases having onset before adulthood [109]. Therefore, future research should account for earlier depressive symptoms to better disentangle temporal ordering and reduce the risk of reverse causation.

Fourth, the ALSPAC cohort comprises mainly white participants (~ 95%) and is not nationally representative. The limited representation of ethnic groups may restrict the generalisability of the findings to other ethnicities [110].

Fifth, attrition in ALSPAC is not completely at random, which may lead to underestimated associations and affect the ability to detect other trajectory classes, such as a limited number of individuals in the high-symptom groups, which can reduce statistical power. To mitigate the effects of missingness, several statistical approaches were carried out, such as inverse probability weighting for logistic regression analyses and FIML for LCGA and path analyses. In addition, variable selection was limited by what was available and practical within the confines of this study, other covariates such as diet and chronic disease are other likely confounders.

Sixth, although IL-6 and CRP are well-established markers of low-grade inflammation, they do not capture the full complexity of inflammatory processes. Other inflammatory markers and pathways not examined in the present study (e.g., tumour necrosis factor-α, interferon-γ) [42, 111] may be differentially associated with sleep disturbances and depressive symptom trajectories.

Seventh, although we adjusted for a range of socio-economic and early-life confounders, there were potentially important covariates we were unable to adjust for, such as preterm birth and anti-depression treatment, and with all observational analyses, there is a risk of residual confounding.

Finally, path analyses were conducted as a secondary, exploratory step to further elucidate potential inflammatory mechanisms underlying the primary associations identified between persistent poor sleep trajectories and persistent higher depressive symptom trajectories. Given this a priori focus, path analyses were not extended to other depressive symptom trajectories, such as persistent moderate or increasing depressive symptoms, as these were not the primary outcomes of interest in the present study. Examination of potential mechanistic pathways for other depressive trajectories remains an important direction for future research.

## Conclusion

Findings from this cohort study indicate that persistent shorter nighttime sleep duration throughout childhood is longitudinally associated with persistent higher depression from adolescence to emerging adulthood. Additionally, IL-6 levels, but not CRP, partially mediated this association. These results emphasise the importance of addressing short sleep duration in childhood, as ongoing sleep issues may be associated with later persistent higher depression risk. The study also highlights the importance of considering early intervention strategies focused on both sleep habits and specific pro-inflammatory markers in children to help prevent depression risk later in life.

## Supplementary Information

Below is the link to the electronic supplementary material.

Supplementary Material 1

## Data Availability

Data was obtained from the ALSPAC. Please note that the study website contains details of all the data that is available through a fully searchable data dictionary and variable search tool: [http://www.bristol.ac.uk/alspac/researchers/our-data/](http:/www.bristol.ac.uk/alspac/researchers/our-data) . Any research team can apply to use the ALSPAC and submit a protocol with an accompanying research question. It is not within the rights of the authors to share this data as it requires individual ALSPAC approvals per researcher.
