# Supplementary material for: Investigating the prospective associations of childhood sleep problems with persistent depression in emerging adulthood: the mediating role of inflammation
Source: Eur Child Adolesc Psychiatry. 2026 May 18;35(9):2865–80. doi: 10.1007/s00787-026-03053-z (PMC13695682; doi:10.1007/s00787-026-03053-z)
Supplement: Supplementary file 1 [file 787_2026_3053_MOESM1_ESM.docx]

**Supplementary Online Content**

**Methods. Additional Information on the distribution of the observed variables included, model specification and treatment of within-class heterogeneity, variance–covariance structure specification, specification and evaluation of trajectory functional forms, and individual trajectories within latent classes**

**Table S1. Factors, time points when they were assessed, and the measurements used**

**Table S2. Differences in socio-demographic variables between non-participating and participating subjects in the study at 12.5 years old**

**Table S3. Multinomial regression of nighttime sleep duration trajectories and depression trajectories.**

## **Table S4. Multinomial regression of MPS trajectories and depression trajectories.**

## **Table S5. Missingness for Nighttime Sleep Duration.**

## **Table S6. Missingness for Midpoint of Sleep.**

## **Table S7. Missingness for SMFQ Depression Symptoms.**

**Table S8 – Adherence to the ‘Guidelines for Reporting on Latent Trajectory Studies’**

**Figure S1. Estimated mean latent class trajectories for night-time sleep duration across adolescence (LCGA).**

**Figure S2. Estimated mean latent class trajectories for midpoint of sleep across adolescence (LCGA).**

**Figure S3. Estimated mean latent class trajectories for depressive symptoms across adolescence (LCGA).**

**References**

**The measurement scale and distributional characteristics of each outcome used in the LCGA models**

The outcomes included in the latent class growth analyses (LCGA) were evaluated with respect to their measurement scale and distributional characteristics. Night-time sleep duration and midpoint of sleep were derived from parent-reported bedtimes and wake-up times and yielded time-based measures that are continuous in nature. Depressive symptoms were assessed using the total score of the Short Mood and Feelings Questionnaire (SMFQ), a 13-item scale with three ordinal response options per item. Although individual SMFQ items are ordinal, the summed total score spans a wide range of values (0–26) and is commonly treated as a quasi-continuous measure in longitudinal studies (e.g., Kingsbury et al., 2016; Knowles et al., 2025; Weavers et al., 2021).

Prior methodological work supports modelling summed ordinal symptom scales as continuous when they comprise multiple items (Bauer & Curran, 2003; Rhemtulla et al., 2012). In large population-based cohorts, including ALSPAC, SMFQ sum scores show good reliability, endorsement across all response categories at each wave, and distributional properties comparable to latent factor scores (Schlechter et al., 2023). Given the scale properties of the SMFQ and its widespread use as a continuous outcome in population-based cohort studies, SMFQ total scores were modelled as continuous outcomes in the LCGA.

Accordingly, all outcomes were modelled as continuous variables in the LCGA. To mitigate the risk of spurious class extraction arising from potential deviations from normality, class enumeration was guided by multiple criteria, including information criteria, entropy, class size, and substantive interpretability.

**Model specification and treatment of within-class heterogeneity**

Alternative specifications of within-class heterogeneity were considered. Specifically, both LCGA and latent growth mixture modelling (LGMM) were considered as alternative specifications for modelling unobserved heterogeneity in nighttime sleep duration, midpoint of sleep, and SMFQ trajectories. LGMM provides greater flexibility by allowing within-class variability in growth parameters (Muthén, 2001), but this flexibility comes at the cost of increased computational complexity, higher sample size requirements, and greater susceptibility to convergence problems and local maxima, particularly in multi-wave population-based data (Muthén, 2006; Twisk & Hoekstra, 2012). In the absence of theoretical justification for estimating within-class variance components and given the study’s emphasis on identifying distinct trajectory shapes rather than quantifying individual-level variation within classes, LCGA was deemed the most appropriate and transparent modelling strategy.

This approach is consistent with prior large-scale developmental studies (Kingsbury et al., 2016; Knowles et al., 2025; Weavers et al., 2021) and aligns with recommendations to prioritise parsimony and interpretability when substantive theory does not dictate within-class variance parameterisation (Jung & Wickrama, 2008).

**Variance–Covariance Structure Specification**

Specification of between-class variance–covariance structures was guided by theoretical considerations, model parsimony, and estimation stability. In line with the LCGA framework, variances and covariances of the growth factors were fixed to zero within classes, such that individual trajectories within each class were assumed to be homogeneous and between-class differences reflected distinct mean developmental patterns (Nagin & Land, 1993; Nagin, 1999). Residual variances of the observed outcomes were constrained to be equal across latent classes, implying comparable within-class variability around the estimated growth curves.

This specification was chosen for several reasons. First, allowing class-specific residual variances or freely estimating variance–covariance matrices substantially increase model complexity and parameter count, which can lead to convergence difficulties and local maxima, particularly in models with multiple repeated measures (Croudace et al., 2003; Jung & Wickrama, 2008; Twisk & Hoekstra, 2012). Second, LCGA is commonly applied in population-based cohort studies as a parsimonious approximation to unobserved heterogeneity, prioritising interpretability of trajectory shapes over modelling within-class variation (Erosheva et al., 2014).

We acknowledge that alternative specifications allowing class-specific residual variances or growth-factor variance–covariance matrices could yield different class solutions. However, given the study aims, sample characteristics, and the risk of spurious class extraction under more flexible parameterisations (Bauer & Curran, 2003), the constrained specification was deemed appropriate. Findings are therefore interpreted as reflecting differences in average developmental trajectories rather than differences in within-class variability.

**Specification and evaluation of trajectory functional forms**

Alternative functional forms of change over time were considered when specifying latent class growth models. For each outcome, models with linear growth trajectories were first estimated, followed by models including quadratic growth terms to allow for non-linear developmental change (Muthén & Shedden, 1999; Jung & Wickrama, 2008). Model comparisons were based on standard fit indices (e.g., Bayesian Information Criterion, Akaike Information Criterion), entropy, class size, and interpretability of the resulting trajectories.

Higher-order polynomial terms were not pursued due to the increased risk of overfitting and instability, particularly in finite mixture models applied to population-based cohort data (Bauer & Curran, 2003). More complex nonlinear growth functions (e.g., logistic or Gompertz curves), semiparametric smoothing approaches, or piecewise models were also considered conceptually but not implemented, as there were no strong theoretical or empirical grounds to expect discontinuities or asymptotic growth patterns across the assessment period, and the number and spacing of waves limited reliable estimation of such models (Grimm & Ram, 2009; Twisk & Hoekstra, 2012).

Accordingly, the final models retained the most parsimonious functional form that adequately captured developmental change while yielding stable and interpretable trajectory classes.

**Individual Trajectories Within Latent Classes**

Plots combining observed individual trajectories with model-estimated mean trajectories were not included. Given the large sample size and number of repeated assessments, displaying individual trajectories would result in severe overplotting, substantially limiting interpretability and obscuring class-level patterns. In addition, the study used population-based cohort data, for which visualisation of individual-level longitudinal profiles raises confidentiality considerations.

Instead, model adequacy and within-class heterogeneity were evaluated using a combination of statistical and substantive criteria, including class-specific trajectory shapes, entropy, posterior classification probabilities, class sizes, and consistency of trajectories with known developmental patterns. Estimated mean trajectories for each latent class are presented to allow clear comparison across classes, which is consistent with prior large-scale latent trajectory studies using LCGA in population cohorts.

# **Table S1. Factors, time points when they were assessed, and the measurements used**

| **Factors** | **Time Points** | **Measurement** |
| --- | --- | --- |
| **Covariates** | | |
| Sex | Birth | Assigned at birth |
| Ethnicity | Birth | We coded ethnicity as white or other, given low cell count across discrete ethnic categories. Other categories included Bangladeshi, Black African, Black Caribbean, Black Other, Chinese, Indian, Pakistani and other ethnic group (not specified) |
| Birth weight, kg | Birth | Weighed in kilograms |
| Age of mother at delivery | Birth | Age |
| Family adversity Index (FAI) | Pregnancy  2 years  4 years | A continuous score measuring cumulative adversity. The FAI measures issues related to housing and family conditions as well as maternal issues such as substance use, affective disorder and involvement in crime. The FAI total score ranges from 0-18, with higher scores indicating more family adversity. |
| Cambridge social interaction and stratification scale (CAMSIS) | Birth | Uses data about occupation to derive social class based and the individuals relative ranking to each other. A higher score indicates higher positions in the social hierarchy such as higher income, educational attainment and stronger connections to those in higher status positions. |
| **Predictors** | | |
| Night-time Sleep Duration | 6months  18months  30 months  3.5 years  4-5 years  5-6 years  6-7 years | Parent-reported item “Time child usually goes to sleep”; “Time child usually gets up”: Night-time sleep duration was extracted from these two measures. Higher scores show longer sleep duration while lower scores shorter sleep duration. |
| Mid-point of sleep (MPS) | 6months  18months  30 months  3.5 years  4-5 years  5-6 years  6-7 years | Parent-reported item Time child usually goes to sleep”; “Time child usually gets up”: MPS was calculated as the middle time point between bedtime and wakeup time and is a proxy of circadian rhythm. A lower score shows a preference for earlier sleep and earlier waking (lark like) whilst a higher score shows a preference for later sleeping (owl like) |
| **Mediators** | | |
| C-Reactive Protein (CRP) | 9 years | Automated particle-enhanced immunoturbidimetric assay (Khandaker et al., 2014). Higher scores indicate elevated levels of CRP in the body. |
| Interleukin-6 (IL-6) | 9 years | Enzyme-linked immunosorbent assay. Higher scores indicate elevated levels of IL-6 in the body. |
| **Outcomes** | | |
| Depression symptomology | 12.5 years  13.5 years  16 years  17.5 years  21 years  22 years | Child self-reported measure of depression via the Short Mood & Feelings questionnaire. A higher score indicates higher depressive symptomology. |

## **Table S2. Differences in socio-demographic variables between non-participating and participating subjects in the study at 12.5 years old**

|  | **Non-participating group in the study** | | **Participating group in the study** | | **Participating versus non-participating** | |
| --- | --- | --- | --- | --- | --- | --- |
|  | *Mean* | *SD* | *Mean* | *SD* | *OR (95% CI)* | *p* |
| Maternal age when born | 26.81 | 5.11 | 29.05 | 4.58 | 1.10 (1.09 to 1.11) | <0.001 |
| Gestational age | 37.24 | 7.46 | 39.45 | 1.85 | 1.14 (1.10 to 1.13) | <0.001 |
| Birth weight, kg | 3.34 | 0.62 | 3.42 | 0.54 | 1.28 (1.21 to 1.35) | <0.001 |
| Family Adversity score | 5.34 | 4.75 | 3.93 | 4.01 | 0.93 (0.92 to 0.94) | <0.001 |
| **Non-participating group in the study Participating group in the study Participating versus non-participating** | | | | | | |
|  | *N* | *%* | *N* | *%* |  |  |
| Sex |  |  |  |  | 0.94 (0.88 to 1.01) | 0.077 |
| Male / Female | 3772 / 3498 | 51.9 / 48.1 | 3919 / 3850 | 50.4 / 49.6 |  |  |
| Ethnicity |  |  |  |  | 0.42 (0.33 to 0.52) | <0.001 |
| White / Other | 4985 / 205 | 96.1 / 3.9 | 7077 / 122 | 98.3 / 1.7 |  |  |
| Preterm delivery |  |  |  |  | 0.56 (0.49 to 0.65) | <0.001 |
| Yes / No | 509 / 3443 | 12.9 / 87.1 | 347 / 4169 | 7.7 / 92.3 |  |  |

*Note:* The individuals associated with attrition at 12.5 years were more frequently born preterm, had lower weight at birth, had shorter gestational age, had younger mothers at birth, had higher scores in family adversity, and were more often of non-white ethnicity.

# **Table S3. Multinomial regression of nighttime sleep duration trajectories and depression trajectories.**

|  | **Persistent higher depression** | | | | **Increasing depression** | | | | **Persistent Moderate depression** | | | |
| --- | --- | --- | --- | --- | --- | --- | --- | --- | --- | --- | --- | --- |
|  | **Unadjusted model** | | **Adjusted model** | | **Unadjusted model** | | **Adjusted model** | | **Unadjusted model** | | **Adjusted model** | |
|  | OR (95%CI) | P value | OR (95%CI) | P value | OR (95%CI) | P value | OR (95%CI) | P value | OR (95%CI) | P value | OR (95%CI) | P value |
| Persistent average longer sleep | Ref |  | Ref |  | Ref |  | Ref |  | Ref |  | Ref |  |
| Persistent short sleep | 2.08 (1.11, 3.89) | 0.022 | 1.99 (1.04, 3.78) | 0.037 | 1.30 (0.78, 2.18) | 0.320 | 1.25 (0.74, 2.12) | 0.399 | 1.38 (0.88, 2.16) | 0.157 | 1.33 (0.85, 2.10) | 0.218 |
| Persistent average short sleep | 1.01 (0.76, 1.34) | 0.959 | 1.08 (0.81, 1.45) | 0.601 | 1.10 (0.92, 1.33) | 0.305 | 1.11 (0.92, 1.34) | 0.298 | 1.02 (0.09, 1.21) | 0.831 | 1.01 (0.85, 1.20) | 0.937 |
| Persistent long sleep | 1.34 (0.98, 1.85) | 0.070 | 1.14 (0.83, 1.59) | 0.421 | 1.20 (0.96, 1.49) | 0.119 | 1.12 (0.90, 1.41) | 0.315 | 0.95 (0.77, 1.18) | 0.662 | 0.93 (0.75, 1.16) | 0.520 |
| Sex assigned at birth^a^ |  |  | 2.84 (2.21, 3.65) | <0.001 |  |  | 2.50 (2.12, 2.95) | <0.001 |  |  | 1.72 (1.49, 1.98) | <0.001 |
| Birth weight, kg |  |  | 1.00 (0.81, 1.24) | 0.986 |  |  | 0.91 (0.80, 1.05) | 0.199 |  |  | 0.93 (0.83, 1.06) | 0.270 |
| Age of mother at delivery |  |  | 0.97 (0.95, 1.00) | 0.048 |  |  | 1.01 (0.99, 1.03) | 0.196 |  |  | 1.02 (1.00, 1.03) | 0.045 |
| Ethnicity^b^ |  |  | 0.64 (0.34, 1.21) | 0.166 |  |  | 1.28 (0.90, 1.82) | 0.164 |  |  | 1.24 (0.90, 1.70) | 0.189 |
| FAI, total score |  |  | 1.09 (1.07, 1.11) | <0.001 |  |  | 1.03 (1.01, 1.05) | 0.001 |  |  | 1.03 (1.01, 1.05) | <0.001 |
| CAMSIS score |  |  | 1.00 (0.99, 1.01) | 0.462 |  |  | 1.00 (0.99, 1.01) | 0.826 |  |  | 1.01 (1.00, 1.01) | 0.008 |
|  |  |  |  |  |  |  |  |  |  |  |  |  |

*Note:* Persistent low depression is the dependent variable reference value. FAI = family adversity index, a higher score. CAMSIS score = social class indicator, larger scores indicate a higher social class/occupational status. ^a^The reference value was male, ^b^the reference value was a white ethnicity.

## **Table S4. Multinomial regression of MPS trajectories and depression trajectories.**

|  | Persistent higher depression | | | | Increasing depression | | | | | Persistent moderate depression | | | | |
| --- | --- | --- | --- | --- | --- | --- | --- | --- | --- | --- | --- | --- | --- | --- |
|  | Unadjusted model | | Adjusted model | | Unadjusted model | | Adjusted model | | | Unadjusted model | | | Adjusted model | |
|  | OR (95%CI) | P value | OR (95%CI) | P value | OR (95%CI) | P value | OR (95%CI) | | P value | OR (95%CI) | | P value | OR (95%CI) | P value |
| Persistent average earlier MPS | Ref |  | Ref |  | Ref |  | Ref |  | | Ref |  | | Ref |  |
| Persistent later MPS | 1.49 (0.90, 2.49) | 0.124 | 1.14 (0.68, 1.93) | 0.621 | 1.14 (0.78, 1.66) | 0.483 | 1.00 (0.68, 1.46) | 0.990 | | 1.28 (0.93, 1.77) | 0.135 | | 1.20 (0.86, 1.67) | 0.291 |
| Persistent earlier MPS | 0.93 (0.68, 1.28) | 0.674 | 1.06 (0.77, 1.46) | 0.735 | 0.91 (0.74, 1.12) | 0.366 | 0.98 (0.79, 1.20) | 0.822 | | 1.02 (0.86, 1.23) | 0.794 | | 1.08 (0.90, 1.30) | 0.408 |
| Persistent average later MPS | 1.33 (1.01, 1.75) | 0.042 | 1.23 (0.93, 1.63) | 0.145 | 1.05 (0.86, 1.27) | 0.647 | 1.00 (0.82, 1.21) | 0.972 | | 0.98 (0.82, 1.17) | 0.846 | | 0.95 (0.80, 1.14) | 0.598 |
| Sex assigned at birth^a^ |  |  | 2.82 (2.20, 3.63) | <0.001 |  |  | 2.49 (2.11, 2.93) | <0.001 | |  |  | | 1.72 (1.49, 1.98) | <0.001 |
| Birth weight, kg |  |  | 1.00 (0.81, 1.24) | 0.968 |  |  | 0.92 (0.80, 1.05) | 0.220 | |  |  | | 0.94 (0.83, 1.06) | 0.283 |
| Age of mother at delivery |  |  | 0.98 (0.95, 1.00) | 0.051 |  |  | 1.01 (1.00, 1.03) | 0.164 | |  |  | | 1.02 (1.01, 1.03) | 0.032 |
| Ethnicity^b^ |  |  | 0.63 (0.33, 1.20) | 0.157 |  |  | 1.30 (0.91, 1.84) | 0.150 | |  |  | | 1.25 (0.91, 1.72) | 0.178 |
| FAI, total score |  |  | 1.09 (1.07, 1.11) | <0.001 |  |  | 1.03 (1.01, 1.05) | 0.001 | |  |  | | 1.03 (1.01, 1.05) | <0.001 |
| CAMSIS score |  |  | 1.00 (0.99, 1.01) | 0.477 |  |  | 1.00 (0.99, 1.01) | 0.875 | |  |  | | 1.07 (1.00, 1.01) | 0.009 |
|  |  |  |  |  |  |  |  |  | |  |  | |  |  |

*Note:* Persistent low depression is the dependent variable reference value. FAI = family adversity index, a higher score. CAMSIS score = social class indicator; larger scores indicate a higher social class/occupational status. ^a^The reference value was male, ^b^the reference value was a white ethnicity.

## Table S5. Missingness for Nighttime Sleep Duration.

| **Time Point (Age)** | **N valid** | **N missing** | **% missing** |
| --- | --- | --- | --- |
| 6 Months | 11221 | 4424 | 28.3% |
| 18 Months | 10852 | 4793 | 30.6% |
| 30 Months | 10033 | 5612 | 35.9% |
| 3.5 years | 9861 | 5784 | 37.0% |
| 4-5 years | 9331 | 6314 | 40.3% |
| 5-6 years | 8437 | 7208 | 46.1% |
| 6-7 years | 8064 | 7581 | 48.4% |

## Table S6. Missingness for Midpoint of Sleep.

| **Time Point (Age)** | **N valid** | **N missing** | **% missing** |
| --- | --- | --- | --- |
| 6 Months | 10971 | 4674 | 29.9% |
| 18 Months | 9281 | 6364 | 40.7% |
| 30 Months | 10001 | 5644 | 36.1% |
| 3.5 years | 9825 | 5820 | 37.2% |
| 4-5 years | 9316 | 6329 | 40.5% |
| 5-6 years | 8428 | 7217 | 46.1% |
| 6-7 years | 8064 | 7581 | 48.4% |

## Table S7. Missingness for SMFQ Depression Symptoms.

| **Time Point (Age)** | **N valid** | **N missing** | **% missing** |
| --- | --- | --- | --- |
| 12.5 years | 6711 | 8934 | 57.1% |
| 13.5 years | 6013 | 9632 | 61.6% |
| 16 years | 4993 | 10652 | 68.1% |
| 17.5 years | 4493 | 11152 | 71.3% |
| 21 years | 3303 | 12342 | 78.9% |
| 22 years | 4021 | 11624 | 74.3% |

**Table S8** – Adherence to the ‘Guidelines for Reporting on Latent Trajectory Studies’

|  | **Checklist Item** | **Reported?** | **Where?** |
| --- | --- | --- | --- |
| **1** | Is the metric of time used in the statistical model reported? | Yes | Statistical analysis- Page 8 |
| **2** | Is information presented about the mean and variance of time within a wave? | Yes | See Table 1 |
| **3a.** | Is the missing data mechanism reported? | Yes | Statistical analysis- Page 8 |
| **3b.** | Is a description provided of what variables are related to attrition/missing data? | Yes | Supplementary  Table S2 |
| **3c.** | Is a description provided of how missing data in the analyses were dealt with? | Yes | Statistical analysis- Page 8 |
| **4** | Is information about the distribution of the observed variables included? | Yes | Supplementary page 2 |
| **5** | Is the software mentioned? | Yes | Statistical analysis- Page 8 |
| **6a.** | Are alternative specifications of within-class heterogeneity considered (e.g., LGCA vs. LGMM) and clearly documented? If not, was sufficient justification provided as to eliminate certain specifications from consideration? | Yes | This is not reported within the manuscript but was explored within the analytic process; see Supplementary page 2 |
| **6b.** | Are alternative specifications of the between-class differences in variance–covariance matrix structure considered and clearly documented? If not, was sufficient justification provided as to eliminate certain specifications from consideration? | Yes | See explanation in Supplementary on page 3 |
| **7** | Are alternative shape/functional forms of the trajectories described? | Yes | See explanation in Supplementary on page 3 |
| **8** | If covariates have been used, can analyses still be replicated? | N/A | No covariates were used |
| **9** | Is information reported about the number of random start values and final iterations included? | Yes | Process by which an optimal solution was derived is explained in Statistical analysis- Page 8 |
| **10** | Are the model comparison (and selection) tools described from a statistical perspective? | Yes | See Results pages 10-12 |
| **11** | Are the total number of fitted models reported, including a one-class solution? | Yes | See Table 2 |
| **12** | Are the number of cases per class reported for each model (absolute sample size, or proportion)? | Yes | See Supplementary S3, S5-S7 |
| **13** | If classification of cases in a trajectory is the goal, is entropy reported? | Yes | See Table 2 |
| **14a.** | Is a plot included with the estimated mean trajectories of the final solution? | Yes | See Figure 1 |
| **14b.** | Are plots included with the estimated mean trajectories for each model? | Yes | See Figure S1-S3 in Supplementary |
| **14c.** | Is a plot included of the combination of estimated means of the final model and the observed individual trajectories split out for each latent class? | No | We cannot report individual data (see explanation in Supplementary on page 3) |
| **15** | Are characteristics of the final class solution numerically described (i.e., means, SD/SE, n, CI, etc.)? | Yes | See Figure 1 for means; sample sizes are specified in results text on pages 10-12 |
| **16** | Are the syntax files available (either in the appendix, supplementary materials, or from the authors)? | Yes | From the authors |

**Figure S1.** Estimated mean latent class trajectories for night-time sleep duration across adolescence (LCGA).

*1-class model*

*2-class model*

*3-class model*

**Figure S2.** Estimated mean latent class trajectories for midpoint of sleep across adolescence (LCGA).

*1-class model*

*2-class model*

*3-class model*

**Figure S3.** Estimated mean latent class trajectories for depressive symptoms across adolescence (LCGA).

*1-class model*

*2-class model*

*3-class model*

**References**

Bauer, D. J., & Curran, P. J. (2003). Distributional assumptions of growth mixture models: Implications for overextraction of latent trajectory classes. Psychological Methods, 8, 338–363. doi:10.1037/1082-989X.8.3.338

Croudace, T. J., Jarvelin, M.-R., Wadsworth, M. E., & Jones, P. B. (2003). Developmental typology of trajectories to nighttime bladder control: Epidemiologic application of longitudinal latent class analysis. American Journal of Epidemiology, 157, 834–842. doi:10.1093/aje/kwg049

Erosheva, E. A., Matsueda, R. L., & Telesca, D. (2014). Breaking bad: Two decades of life-course data analysis in criminology, developmental psychology, and beyond. Annual Review of Statistics and Its Application, 1, 301–332.

Jung, T., & Wickrama, K. (2008). An introduction to latent class growth analysis and growth mixture modeling. Social and Personality Psychology Compass, 2(1), 302–317. doi:10.1111/j.1751-9004.2007.00054.x

Kingsbury, M., Weeks, M., MacKinnon, N., Evans, J., Mahedy, L., Dykxhoorn, J., & Colman, I. (2016). Stressful life events during pregnancy and offspring depression: evidence from a prospective cohort study. Journal of the American Academy of Child & Adolescent Psychiatry, 55(8), 709-716.

Knowles, G., Stanyon, D., Yamasaki, S., Miyashita, M., Gayer-Anderson, C., Endo, K., ... & Nishida, A. (2025). Trajectories of depressive symptoms among young people in London, UK, and Tokyo, Japan: a longitudinal cross-cohort study. The Lancet Child & Adolescent Health, 9(4), 224-233.

Muthén, B. (2001). Latent variable mixture modeling. In Marcoulides, G., & Schumacker, R. (Eds.), New developments and techniques in structural equation modeling (pp. 1–33). Mahwah, NJ: Lawrence Erlbaum.

Nagin, D. S., & Land, K. C. (1993). Age, criminal careers, and population heterogeneity: Specification and estimation of a nonparametric, mixed Poisson model*. Criminology, 31, 327–362. doi:10.1111/crim.1993.31.issue-3

Nagin, D. S. (1999). Analyzing developmental trajectories: A semi-parametric group based approach. Psychological Methods, 4, 139–157. doi:10.1037/1082-989X.4.2.139

Rhemtulla, M., Brosseau-Liard, P. É., & Savalei, V. (2012). When can categorical variables be treated as continuous? A comparison of robust continuous and categorical SEM estimation methods under suboptimal conditions. Psychological Methods, 17(3), 354–373. https://doi.org/10.1037/a0029315

Schlechter, P., Wilkinson, P. O., Ford, T. J., & Neufeld, S. A. (2023). The Short Mood and Feelings Questionnaire from adolescence to emerging adulthood: Measurement invariance across time and sex. Psychological Assessment, 35(5), 405.

Twisk, J., & Hoekstra, T. (2012). Classifying developmental trajectories over time should be done with great caution: A comparison between methods. Journal of Clinical Epidemiology, 65, 1078–1087. doi:10.1016/j.jclinepi.2012.04.010

Weavers, B., Heron, J., Thapar, A. K., Stephens, A., Lennon, J., Jones, R. B., ... & Rice, F. (2021). The antecedents and outcomes of persistent and remitting adolescent depressive symptom trajectories: a longitudinal, population-based English study. The Lancet Psychiatry, 8(12), 1053-1061.
